# Efficacy and Outcomes of the Hormone-Releasing Levonorgestrel-Intrauterine System for Adenomyosis Treatment

**DOI:** 10.7759/cureus.97669

**Published:** 2025-11-24

**Authors:** Vibisha Pragash, Alpa Khakhar

**Affiliations:** 1 Obstetrics and Gynaecology, Apollo Hospitals, Chennai, IND; 2 Urogynaecology, Apollo Hospitals, Chennai, IND

**Keywords:** adenomyosis, dysmenorrhea, heavy menstural bleeding, lng-ius, mirena coil

## Abstract

Background

Adenomyosis refers to the presence of endometrial tissues within the uterine myometrium. The main presenting features are dyspareunia, dysmenorrhea, heavy menstrual bleeding (HMB), and chronic pelvic pain (CPP). With an increasing prevalence, symptomatic management is becoming more necessary. This study aimed to assess the efficacy of symptomatic relief and possible causes of failure six months after levonorgestrel-intrauterine system (LNG-IUS) insertion.

Methodology

This prospective, observational study was conducted over six months involving 110 patients who underwent LNG-IUS intrauterine insertion at a tertiary healthcare center and met the eligibility criteria. Pain assessment was performed using the Visual Analog Scale (VAS). HMB was assessed using the Pictorial Blood Assessment Chart, and possible causes of failure were analyzed based on uterocervical length (UCL) and uterine volume measurements.

Results

The VAS score before and six months after insertion of LNG-IUS was from 5.54 ± 3.10 to 1.37 ± 2.53 for dysmenorrhea, 1.75 ± 2.57 to 0.50 ± 1.57 for dyspareunia, and 1.58 ± 2.24 to 0.76 ± 1.99 for CPP. Blood loss assessment (in mL) before and after insertion was 66.73 ± 36.82 to 4.86 ± 12.88. All four assessments had p-values <0.001 and were significant. Those with significantly increased UCL and increased uterine volume had a cumulative displacement or expulsion rate of 4.54%.

Conclusions

This study demonstrated that LNG-IUS is highly effective in providing symptomatic relief to patients with adenomyosis. The overall satisfactory rate was 87.3%, and the failed outcome rate was 10.9%.

## Introduction

Adenomyosis, also known as endometriosis interna, is the presence of endometrial tissues (glandular and stromal components) within the myometrium of the uterus [1-3]. It is a benign invasion causing a diffuse enlargement of the uterus. The incidence of adenomyosis remains unknown, with its prevalence varying greatly due to a lack of proper standardized definitions and criteria [4,5]. In India, its prevalence likely ranges from 6% to 55% in hysterectomy specimens [4]. Diagnostic modalities for adenomyosis include a patient’s medical history with presenting symptoms, clinical examination, and imaging techniques. With the latest imaging techniques, more cases of adenomyosis are easily identified. Few studies have found a higher prevalence of 24% with the help of transvaginal ultrasound [6-8]. However, in the general population, it is known to be more common among the elderly reproductive age group (fourth and fifth decades of life). Although occasionally adenomyosis can be noted in younger reproductive years with infertility as the complaint, the average age of symptomatic women is usually around 40 years. It is also known to be more common among parous women [1,3].

The most common presenting features include abnormal uterine bleeding (AUB), dysmenorrhea, chronic pelvic pain (CPP), and dyspareunia [1,3]. AUB in this scenario refers to heavy menstrual bleeding (HMB), which is the most common presenting feature. Treatment is becoming more than necessary for these patients [9]. Here, CPP refers to lower abdominal or pelvic pain lasting for more than six months, and dysmenorrhea refers to painful menstrual cycles. Adenomyosis is known to have hormonal and enzymatic discrepancies [3,10].

Treatment can vary from medical management (as the first line of management), surgical management, and other novel techniques. Medical management can include hormonal and non-hormonal treatments. Non-hormonal treatment is for symptomatic management, whereas hormonal treatment requires the use of oral medications regularly. Patients may forget to take their medications on a routine basis for six to nine months, whereas with the insertion of a hormone-releasing levonorgestrel-intrauterine system (LNG-IUS), which has validity of up to five years, it proves to be very effective [11,12]. In the past, hysterectomy was the only way to manage adenomyosis, but with current improvements in the medical field, the emergence of LNG-IUS has revolutionized the treatment modality. With various risk factors ranging from menstrual abnormalities, medications, parity, uterine surgeries, to even tissue injury and repair mechanisms, displaced Müllerian cells, and, finally, genetic causes [1-3,10,13-18]. With known causes and risk factors, the main aim of the medical treatment is to reduce the production of endogenous estrogen or to induce endometrial tissue stabilization with progesterone. LNG-IUS makes this possible. Hormone-releasing LNG-IUS insertion into the uterus causes decidualization and stabilization of the ectopic endometrial tissue.

This study aimed to determine the efficacy of LNG-IUS insertion in symptomatic improvement of dysmenorrhea, AUB (HMB), dyspareunia, and CPP experienced by patients with adenomyosis six months after insertion. The primary objective was to determine the degree of symptomatic improvement or resolution. The secondary objective was to assess the possible reasons for failure.

## Materials and methods

This prospective, observational study was conducted from January 2023 to June 2024 at a tertiary health care facility. All women who presented to the Outpatient Department (OPD) and/or Inpatient Department (IPD) of the the hospital and fulfilled the inclusion and exclusion criteria were included in this study. After obtaining clearance from the Institutional Ethics Committee and the scientific committee (approval number: AMH-DNB-017/02-23), the study was conducted at a tertiary healthcare facility, with informed consent obtained from women willing to participate, as well as their attendants. In need of a translator, their signatures were also obtained.

Inclusion and exclusion criteria

We included all parous women who accepted LNG-IUS as their treatment modality, aged between 35 and 45 years, and were diagnosed with adenomyosis based on the history, clinical examination, and ultrasound of the pelvis, with the Morphological Uterus Sonographic Assessment, being followed by a single sonologist. Those aged less than 35 years were excluded in view of mainly presenting infertility as their complaint. We excluded known cases of adenomyosis who were on other medical and surgical management techniques, women in the age group of 35-45 years who had fibroids measuring more than 3 cm and pelvic endometriosis, those with contraindications to LNG-IUS, pregnant women, and those with endometrial pathologies.

Methodology

Women aged 35-45 years were included in this study. After ruling out pregnancy, all women who met the inclusion/exclusion criteria were included in this observational study. Before LNG-IUS insertion, all patients underwent endometrial sampling by means of Pipelle curettes on an outpatient basis and endometrial curettage on an inpatient basis. Endometrial malignancies were ruled out for all patients. LNG-IUS was inserted into the uterine cavity during the menstrual cycle with necessary precautionary measures, preferably within seven days of the onset of menstruation. If inserted beyond seven days, a barrier method of contraception or sexual abstinence for the next seven days was advised. With an empty bladder, a bimanual examination was performed to estimate the size and position of the uterus. Under strict aseptic precautions, LNG-IUS insertion was performed. The data were collected with the help of a questionnaire designed by the primary author, containing a total of 10 questions, of which five were asked before the insertion of LNG-IUS, and the remaining five after the insertion of LNG-IUS (Appendices).

The questionnaire included a pain assessment scale (Visual Analog Scale (VAS) score) for dysmenorrhea, dyspareunia, and CPP. The scores ranged from 0 to 10, where 0 denoted no pain and 10 denoted the worst pain (1-3: mild pain, 4-6: moderate pain, 7-10: severe pain). Patients chose the degree of pain they perceived. This was done before and after LNG-IUS insertion. Blood loss assessments were done using two methods: (1) the Pictorial Blood Assessment Chart (PBAC). The chart was given to women with AUB, and scoring was done. Normal scoring was ≤100, and HMB had a score of >100. In PBAC, pads were given a score as the number of pads/day, with the number of days of menstrual flow. A pictorial representation of the staining of the pads was in the PBAC chart. Lightly stained pads were given 1 point for each, medium-stained pads were given 5 points for each, and heavily stained pads were given 20 points for each. (2) The red blood capacity of modern menstrual pads was calculated as per the study conducted by DeLoughery et al., with mild flow denoted as 4 mL, moderate flow as 15.5 mL, and heavy flow as 31 mL of blood [19]. Blood spotting ranged from 10-20 drops, denoting 0.5 to 1 mL [20]. Ultrasound-guided uterine volume assessment was calculated as follows: 0.52 × uterocervical length (UCL) × anteroposterior diameter (APD) × transverse diameter. Average adults have a UCL of 7.5 cm, APD of 2.5 cm, and transverse diameter of 5 cm [21]. The results were noted in Excel format, followed by statistical analysis of their efficacy.

Statistical analysis

Following a convenient sampling method, the sample size was calculated to be 110 based on the following formula: N = z²pq/d², with prevalence from a previous study of 24% for adenomyosis and clinical allowable error (d) of 8% [8]. Summary statistics are presented as mean ± SD and frequency (percentage) for the continuous and categorical factors, respectively. The median (interquartile range) was presented while the data were skewed. Student’s t-test or Mann-Whitney U test was used to determine significant differences between before and after insertion. The chi-square or Fisher’s exact test was used to determine the association between two independent categorical factors. McNemar’s-Bowker test was used to determine the proportion changes in the PBAC before and after insertion. P-values <0.05 were considered statistically significant. All statistical analysis was performed using SPSS version 28.0 (IBM Corp., Armonk, NY, USA).

## Results

Demographic analysis for age, body mass index, and mode of delivery resulted in insignificant p-values >0.05 (Table 1).

**Table 1:** Demographic analysis. ^#^: Student’s t-test; ^*^: Fisher’s exact test. The table presents a comparative demographic profile of participants based on treatment outcomes (successful vs. failed). Variables include age, body mass index (BMI), and mode of delivery. Age and BMI were analyzed using Student’s t-test; mode of delivery was compared using Fisher’s exact Test. There were no statistically significant differences between the two groups, suggesting demographic variables were not confounding factors in treatment outcomes.

Variable	Successful outcome (n = 98)	Failed outcome (n = 12)	P-value
Age (in years), mean ± SD	38.8 ± 3.48	40.67 ± 4.10	0.092^#^
BMI, mean ± SD	26.0 ± 3.82	26.61 ± 3.03	0.300^#^
Mode of delivery			
Vaginal delivery	38	3	0.500^*^
Lower-segment cesarean section	60	9	

All 110 patients were followed up after six months, and the following results were obtained regarding the four criteria of dysmenorrhea, dyspareunia, CPP, and blood loss assessment. The prevalence of dysmenorrhea in this study was 89.09%, totalling 98 patients with symptoms ranging from mild to severe Pain. After six months of follow-up, following the insertion of LNG-IUS, 80.61% had an overall pain alleviation. No improvement in symptoms was noticed in 13 patients, and worsening of symptoms was noticed in one patient. The prevalence of dyspareunia in this study was 43.68%, totalling 48 patients with symptoms ranging from mild to severe pain. After six months of follow-up following the insertion of LNG-IUS, 75.0% had an overall pain alleviation. No improvement in symptoms was noticed in nine patients, and worsening of symptoms was noticed in three patients. Dryness of the vagina was noticed in five patients. The prevalence of CPP in this study was 44.54%, totalling 49 patients with symptoms ranging from mild to severe pain. After six months of follow-up following the insertion of LNG-IUS, 75.51% had an overall pain alleviation. No improvement in symptoms was noticed in six patients, and worsening of symptoms was noticed in nine patients (Tables 2-4).

**Table 2:** Pain category assessment for dysmenorrhea, dyspareunia, and CPP before and after LNG-IUS insertion. The table summarizes the distribution of pain severity categories (based on VAS score) before and after LNG-IUS insertion for three conditions: dysmenorrhea, dyspareunia, and CPP. Pain levels were categorized into no, mild, moderate, and severe pain. “Failed outcomes” indicate patients whose symptoms did not improve post-insertion. This descriptive analysis aids in visualizing shifts in pain severity. LNG-IUS = levonorgestrel-intrauterine system; CPP = chronic pelvic pain; VAS = Visual Analog Scale

Pain category (VAS score)		No pain	Mild pain	Moderate pain	Severe pain	Total	Failed outcomes
Dysmenorrhea	Before insertion	12	22	23	53	110	-
	After insertion	64	28	4	9	110	5
Dyspareunia	Before insertion	62	28	8	12	110	-
	After insertion	88	12	2	3	110	5
CPP	Before insertion	61	33	11	5	110	-
	After insertion	86	11	3	6	110	4

**Table 3:** Symptomatic improvement after the six-month follow-up for dysmenorrhea, dyspareunia, and CPP. This table outlines specific shifts in pain categories (from severe/moderate/mild to lower categories) post LNG-IUS insertion, showing improvement percentages for dysmenorrhea, dyspareunia, and CPP. Each row denotes a type of symptomatic relief experienced. Percent improvement is calculated based on the total number of patients with pre-existing pain in each category. The data supports significant pain reduction across all three domains. LNG-IUS = levonorgestrel-intrauterine system; CPP = chronic pelvic pain

Before to after insertion of LNG-IUS (patients benefited)		Number of patients	Total	Improvement assessment in %
Dysmenorrhea	Mild pain to no pain	14	52	53.06
	Moderate pain to no pain	14		
	Severe pain to no pain	24		
	Moderate pain to mild pain	8	23	30.26
	Severe pain to mild pain	15		
	Severe pain to moderate pain	4	4	7.55
Dyspareunia	Mild pain to no pain	19	31	64.58
	Moderate pain to no pain	6		
	Severe pain to no pain	6		
	Moderate pain to mild pain	1	5	25
	Severe pain to mild pain	4		
	Severe pain to moderate pain	0	0	0
CPP	Mild pain to no pain	23	32	65.31
	Moderate pain to no pain	7		
	Severe pain to no pain	2		
	Moderate pain to mild pain	3	5	31.25
	Severe pain to mild pain	2		
	Severe pain to moderate pain	0	0	0

**Table 4:** Overall symptomatic assessment for dysmenorrhea, dyspareunia, and CPP. ^*^: Student’s t-test/Mann Whitney U test. Boldface indicates statistical significance. ^#^: Chi-square/Fisher’s exact test. Boldface indicates statistical significance. This table presents both numerical pain score data (mean ± SD, median, range) and categorical data for dysmenorrhea, dyspareunia, and CPP, before and after LNG-IUS insertion. The statistical significance of symptom reduction is shown using Student’s t-test or Mann-Whitney U test for continuous variables, and chi-square or Fisher’s Exact test for categorical data. Significant improvements were noted in all domains (p < 0.001), confirming the effectiveness of LNG-IUS in pain relief. LNG-IUS = levonorgestrel-intrauterine system; CPP = chronic pelvic pain

Overall symptomatic assessment		Insertion, n (%)		P-value*
		Before	After	
Dysmenorrhea	Pain score^*^			<0.001
	Mean ± SD	5.54 ± 3.10	1.37 ± 2.53	
	Median (IQR)	6 (2–8)	0 (0–2)	
	Range	0–10	0–10	
	Pain categories^#^			<0.001
	No pain	12 (10.9)	64 (58.2)	
	Mild pain	22 (20.0)	28 (25.5)	
	Moderate pain	23 (20.9)	4 (3.6)	
	Severe pain	53 (48.2)	9 (8.2)	
	Failed outcome	-	5 (4.5)	
Dyspareunia	Pain score^*^			<0.001
	Mean ± SD	1.75 ± 2.57	0.50 ± 1.57	
	Median (IQR)	0 (0–2)	0 (0–0)	
	Range	0–8	0–9	
	Pain categories^#^			<0.001
	No pain	62 (56.4)	88 (80.0)	
	Mild pain	28 (25.5)	12 (10.9)	
	Moderate pain	8 (7.3)	2 (1.8)	
	Severe pain	12 (10.9)	3 (2.7)	
	Failed outcome		5 (4.5)	
CPP	Pain score^*^			<0.001
	Mean ± SD	1.58 ± 2.24	0.76 ± 1.99	
	Median (IQR)	0 (0–2)	0 (0–0)	
	Range	0–8	0–8	
	Pain categories^#^			<0.001
	No pain	61 (55.5)	86 (78.2)	
	Mild pain	33 (30)	11 (10)	
	Moderate pain	11 (10)	3 (2.7)	
	Severe pain	5 (4.5)	6 (5.5)	
	Failed outcome		4 (3.6)	

The prevalence of HMB in this study was found to be 74.54%, totalling 82 patients, with 61 patients experiencing alleviation of symptoms, amounting to 74.39%. Among these, 15 patients had complete amenorrhea (no flow: menstrual cycle stopped/amenorrhea), amounting to 18.29%. Overall, 13 patients had a PBAC >100 after insertion in view of spotting to mild flow, with the number of days ranging from 3 to persistent. Two patients had no improvement in symptoms (Tables 5-7).

**Table 5:** Assessment of patients with HMB and normal flow. This table displays the change in PBAC scores among patients who initially presented with HMB (PBAC >100) or normal menstrual flow (PBAC ≤100). Post-insertion outcomes include complete cessation of flow, reduction to normal flow, persistence of HMB, and failed treatment. The data provides insight into LNG-IUS effectiveness in reducing menstrual blood loss. LNG-IUS = levonorgestrel-intrauterine system; PBAC = Pictorial Blood Assessment Chart; HMB = heavy menstrual bleeding

PBAC score analysis	No flow – menstrual cycle stopped	>100	≤100	Failed outcome	Total
Before insertion – PBAC score >100 (HMB), after insertion their outcomes	15	15	46	6	82
Before Insertion – PBAC score ≤100 (normal flow), after insertion their outcomes	12	1	14	1	28

**Table 6:** Blood loss assessment using PBAC score. ^^^^: McNemar’s-Bowker test. This table displays the change in PBAC scores among patients who initially presented with HMB (PBAC >100) or normal menstrual flow (PBAC ≤100). Post-insertion outcomes include complete cessation of flow, reduction to normal flow, persistence of HMB, and failed treatment. The data provides insight into LNG-IUS effectiveness in reducing menstrual blood loss. LNG-IUS = levonorgestrel-intrauterine system; PBAC = Pictorial Blood Assessment Chart; HMB = heavy menstrual bleeding

Before insertion	After insertion, n (%)				Overall	P-value^^^^
	No flow	Normal flow	HMB	Failed outcome		
Normal flow	12 (10.9)	14 (12.7)	1 (0.9)	1 (0.9)	28 (25.5)	0.058
HMB	15 (13.6)	46 (41.8)	15 (13.6)	6 (5.5)	82 (74.5)	
Total	27 (24.5)	60 (54.5)	16 (14.5)	7 (6.3)	110	

**Table 7:** Blood loss assessment. ^*^: Student’s t-test/Mann Whitney U test. Boldface indicates statistical significance. Quantitative assessment of menstrual blood loss (in mL and pads/day) was performed before and after LNG-IUS insertion using both PBAC and patient-reported data. Statistical comparisons (Student’s t-test or Mann-Whitney U test) show significant reductions in mean blood loss volume and pad usage (p < 0.001), supporting the efficacy of LNG-IUS in reducing menstrual bleeding. LNG-IUS = levonorgestrel-intrauterine system; PBAC = Pictorial Blood Assessment Chart

Blood loss assessment		Insertion, n (%)		P-value*
		Before	After	
Blood loss, mL	Mean ± SD	66.73 ± 36.82	4.86 ± 12.88	<0.001
	Range	19.5–159.5	0–78	
Flow	Flow (Pads/Day)			<0.001
	Median (IQR)	4.5 (3–6)	1 (0–2)	
	Range	1–15	0–24	
	Final			<0.001
	Median (IQR)	62.25 (31–94)	0.5 (0–4.5)	
	Range	19.5–159.5	0–78	

Among the 12 failed outcome patients, two had displacement of LNG-IUS, and three had expelled it. The three patients who had expulsion of LNG-IUS were found to have severe adenomyosis with an average uterine volume of 240.7 mL. The average UCL for displaced LNG-IUS was found to be 8.99 cm. The cumulative assessment of failed outcomes (displacement and expulsion) with successful outcomes is presented in Table 8.

**Table 8:** Analysis of UCL and uterine volume. ^#^: Student’s t-test. Boldface indicates statistical significance. This table compares UCL and uterine volume between patients with successful outcomes and those with device displacement/expulsion. Data were analyzed using Student’s t-test. Patients with failed outcomes had significantly greater UCL and uterine volume (p < 0.001 and p = 0.017, respectively), suggesting anatomical factors may predict LNG-IUS expulsion risk. UCL = uterocervical length; LNG-IUS = levonorgestrel-intrauterine system

Variable	Successful outcome (n = 98)	LNG-IUS displacement or expulsion (n = 5)	P-value^#^
UCL (in cm), mean ± SD (range)	7.04 ± 1.05 (5.224–11)	9.43 ± 0.60 (8.36–9.78)	<0.001
Uterine volume, mean ± SD (range)	82.46 ± 31.19 (31.09–236.53)	197.50 ± 65.26 (104.82–270.90)	0.017

On follow-up after six months, 65 patients had irregular bleeding patterns, which ranged from spotting to mild flow, and two patients retained HMB. Two patients had a displaced LNG-IUS, and one patient had a mild pelvic infection and was medically managed. Overall, 12 people out of 110 (10.9%) had failed outcomes. Seven patients experienced the adverse effects of LNG-IUS with cramping pelvic pain. The overall satisfaction rate was found to be 87.3% (96 out of 110). In total, 27 patients had no flow (menstrual cycle stopped) after a follow-up of six months (24.5%), and necessary tests were done to rule out pregnancy. Among these 27 patients, only one patient with severe adenomyosis had complete resolution. There were no pregnancies/ectopic pregnancies, hypersensitivity reactions, perforations, embedment, or simple ovarian cyst formation.

**Figure 1:**
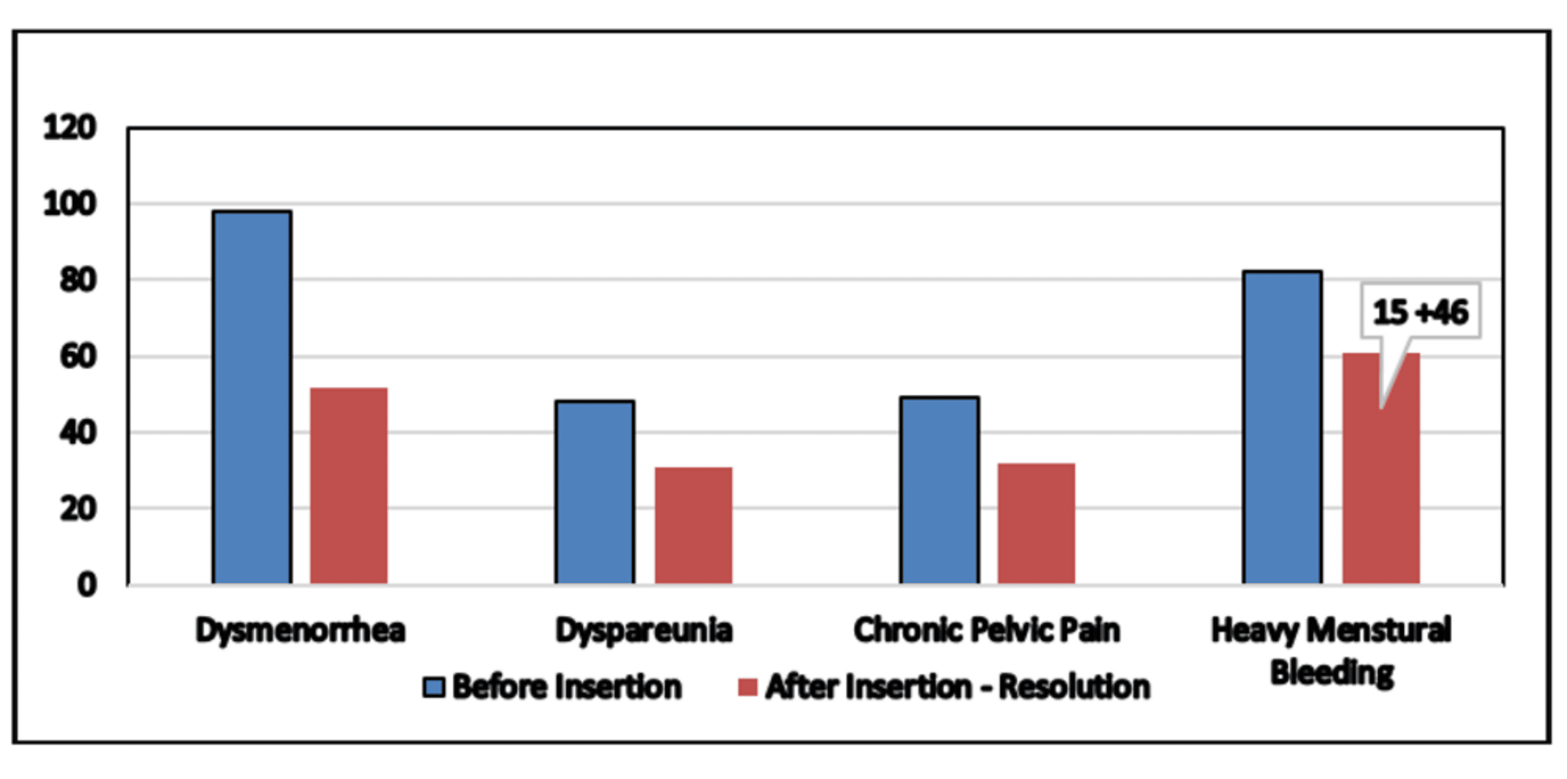
Resolution of the symptoms. After insertion of LNG-IUS, HMB was reported in 61 patients (mentioned in the figure as “15+46”), referring to 15 patients with amenorrhea and 46 patients with normal flow. After insertion of LNG-IUS, the number of patients with a VAS score of 0 (no pain) has been shown for dysmenorrhea, dyspareunia, and CPP. LNG-IUS = levonorgestrel-intrauterine system; HMB = heavy menstrual bleeding; VAS = Visual Analog Scale; CPP = chronic pelvic pain

Overall, there was significant pain alleviation for dysmenorrhea, dyspareunia, and CPP (p < 0.001). Moreover, there was a significant reduction in the number of pads/day and average blood loss (p < 0.001). A p-value of 0.058 denotes the improvement of HMB; even though it is more than 0.05, it is still significant when considering the lower degrees of soakage. Significance was also noted in UCL and uterine volume, with both having p-values <0.05. The overall symptomatic improvements are presented in Figure 1.

## Discussion

This study aimed to determine the outcomes regarding the symptoms of dysmenorrhea, CPP, dyspareunia, and AUB (HMB) after a six-month follow-up period post-LNG-IUS insertion. This study was able to determine the degree of improvement/resolution of symptoms. It was also able to determine the possible reasons for failure, thereby meeting both the primary and secondary objectives of the study.

Menorrhagia (HMB) and dysmenorrhea have long been considered the classic symptoms of adenomyosis, with the prevalence of dysmenorrhea/pelvic pain ranging between 38% and 87% [3,22]. In this study, the prevalence of dysmenorrhea and CPP was found to be 89.09% and 44.54%, respectively. The prevalence of menorrhagia (HMB) ranges from 26% to 49% [23,24]. In this study, the prevalence of HMB was found to be 74.54%.

The presence or absence of dyspareunia is frequently not questioned or recorded by clinicians [22]. A study by Yu et al. (2020) reported a detailed assessment of the incidence of adenomyosis along with symptomatic analysis [25]. Their study did not include the individual prevalence of dyspareunia. This study, however, included the prevalence of dyspareunia (43.68%).

Treatment is becoming more than necessary for patients. Just symptomatic management with analgesics and anti-heavy flow medications will become inadequate. This study has shown dramatic pain alleviation for dysmenorrhea, dyspareunia, and CPP. Complete pain alleviation was seen to be 53.06% for dysmenorrhea, 64.58% for dyspareunia, and 65.31% for CPP. Attainment of amenorrhea (no flow) occurred in 24.5% of patients. Other similar studies have shown improvement in VAS score, uterine volume, and blood loss, thereby justifying the use of adenomyosis as a long-term remedy [26,27]. Mansukhani et al. showed that 27.5% of patients had amenorrhea during the 18-month follow-up [28]. The above study mentioned an overall satisfaction of 80%. Irregular bleeding patterns, which ranged from spotting to mild flow, were a common side effect of progesterone-only treatment [12,26].

Sheng et al. reported an overall satisfaction of 72.5% during a three-year follow-up period [26]. This study showed that the overall satisfaction rate for LNG-IUS was 87.3%. This study showed improvement in VAS score for dysmenorrhea from 5.54 ± 3.10 to 1.37 ± 2.53 (p < 0.001). Shaaban et al. reported VAS score improvement of 6.23 ± 0.67 to 1.68 ± 1.25 [27], Sheng et al. reported a VAS score improvement from 77.9 ± 14.7 to 11.8 ± 17.9 after 36 months [26], and Lin et al. reported that in their interventional group (with LNG-IUS) VAS score was 6.5 ± 2.5 vs. 4.1 ± 3.6 after 12 months of follow-up and 6.1 ± 2.7 vs. 3.7 ± 3.7 after 24 months of follow-up [29]. Morelli et al. reported a VAS score for pain alleviation for pelvic pain in 49.3% of patients. This study has shown that 75.51% (from 1.58 ± 2.24 to 0.76 ± 1.99 with a p<0.001) had improvement [30].

In the study by Shaaban et al., during the six-month follow-up, the average number of bleeding days per month was 2.63 (p < 0.001), the average number of sanitary pads used per day was 2 (p < 0.001), and the average number of blood-free days was 25.39 (p < 0.001) [27]. In this study, the average number of pads per day was found to reduce from 4.5 to 1 (p < 0.001), with complete amenorrhea achieved in 24.5%.

As recommended by the National Institute for Health and Care Excellence guidelines (2018), this study does prove to be very efficient in the management of adenomyosis, thereby proving that LNG-IUS can be preferred as the first line of medical management for adenomyosis treatment [31]. Patients who desire contraception and are unwilling to undergo extensive surgical management can opt for LNG-IUS (SOGC Grade A, Level I) [32]. This study recommends the usage of LNG-IUS in the management of adenomyosis in those with a uterine volume <197.50 mL and a UCL <9.43 cm.

As the most commonly available LNG-IUS is Mirena, a more detailed understanding of it becomes necessary. Trials have shown adverse events in Mirena users, including abdominal pain (5%) for those who used it for more than five years. This study has shown the development of pelvic pain in 3.63%. Trials have also reported adverse events in 5% of Mirena users, including dysmenorrhea (6.4%), libido decrease, and vulvovaginitis was reported in 10.5%. Amenorrhea develops in approximately 20% of Mirena users by the end of year one [12].

This study has shown a worsening in dysmenorrhea for 0.91% and overall amenorrhea achieved after six months was 24.5%. This study has also shown dyspareunia in view of vaginal dryness in 1.81% and only 0.91% had had a pelvic infection. This study has shown zero allergic reactions.

There was no pregnancy reported in this study. The added advantage is that once pregnancy has been ruled out in amenorrheic patients, pregnancy tests need not be repeatedly done unless absolutely required, for example, if the patient shows signs of pregnancy or ectopic pregnancy [12,33-35]. However, further analysis in other trials has shown the following data: with Mirena, the reported 12-month pregnancy rates were ≤0.2 per 100 women (0.2%) and the accumulated five-year pregnancy rate was approximately 0.7 per 100 women (0.7%). The pregnancy rate as per the Pearl Index at the end of the sixth year was found to be 0.34; one pregnancy occurred during the sixth year, within seven days of Mirena’s removal.

In this study, only one patient had vaginal discharge. However, on comparison of different LNG-IUS devices available, genital or vaginal discharge occurred in the following percentages of patients receiving various LNG-IUS: Mirena 14.9%, Kyleena 4.5%, Liletta 5.8%, and Skyla 4.2%.

Dyspareunia was reported in fewer than 5% of patients (Mirena), 9.6% of patients (Liletta), and was not specifically reported for Skyla or Kyleena but estimated at less than 1% [12,33-35]. This study demonstrated alleviation in dyspareunia to be (VAS Score) from 1.75 ± 2.57 to 0.50 ± 1.57 (p < 0.001).

Pelvic pain has been reported in users of LNG-IUS in clinical trials: 22.6% for Mirena, 8.7% for Liletta, 6.2% to 18.9% for Skyla, and 8.2% to 21% for Kyleena [12,33-35]. This study has depicted an improvement in chronic pelvic pain (VAS score from 1.58 ± 2.24 to 0.76 ± 1.99; p< 0.001) and with a worsening of pelvic pain to 8.18%.

Hysterectomy should be the last resort in the treatment of adenomyosis in those who are willing to undergo a final solution. LNG-IUS should be the first-line treatment, given that its overall satisfaction rate is high with avoidance of a major surgery [36].

This study showed an expulsion rate of 2.73% occurring only in severe adenomyosis patients with an average uterine volume of 240.7 mL. Normal uterine volume ranges from 15 to 56 mL. In purely adenomyosis of the uterus, the maximum size of the uterus will be 12 weeks, which can have a maximum volume of 240 mL [21,37]. This study also showed a displacement rate of 1.82% in patients with an average UCL of 8.99 cm. With an overall assessment, those with significantly increased UCL (average 9.43 cm) and increased uterine volume (average 197.50 mL), there is a cumulative displacement or expulsion rate of 4.54%. Therefore, in patients who have an increased uterine volume or increased UCL, there is a high chance of LNG-IUS displacement or expulsion.

However, multiple studies have shown that the LNG-IUS can be sutured into the uterine cavity with promising results [38-41]. Liang et al. noted improvements in pregnancy/ clinical pregnancy and implantation rates among women undergoing assisted reproductive technologies who were suffering from adenomyosis and were treated with LNG-IUS [42]. Lin et al. investigated the efficacy of LNG-IUS in the prevention of recurrence of postoperative adenomyotic symptoms. In their study, after a 24-month follow-up, a significant reduction was noted in dysmenorrhea with improvement in hemoglobin levels (with p < 0.05) [29]. In a study conducted in China by Xu et al., which involved hysteroscopic-guided LNG-IUS uterine suture fixation for adenomyosis, the expulsion rate had a significant p-value <0.05, with no other different outcomes when compared to the conventional LNG-IUS uterine insertion method [38]. In the study conducted in China by Lv et al. regarding the feasibility and effectiveness of hysteroscopic suture fixation of LNG-IUS for adenomyosis, the VAS, PBAC score, and cancer antigen 125 (CA-125) were significantly reduced (p < 0.001), with a conclusion of having a low expulsion rate with significant improvement in dysmenorrhea and bleeding [39]. Therefore, patients who suffer from severe dysmenorrhea with increased UCL and high uterine volume are at risk of displacement/expulsion of LNG-IUS, and can be treated with intrauterine suture fixation of LNG-IUS.

This study has some limitations. The major limitation is its single-center design. Further, markers such as CA-125 could have been measured before and six months after the intrauterine insertion of LNG-IUS.

## Conclusions

In adenomyosis, with the endometrial tissues situated in the wrong place within the myometrium, undergoing monthly hormonal changes, and the blood having no place to escape, leads to symptoms. In the past, it was difficult to diagnose adenomyosis; however, with the emergence of the latest imaging technologies, the detection of adenomyosis has significantly increased. With dysmenorrhea and HMB being the main complaints, which can affect the day-to-day life of the individual, it is becoming necessary to treat them. This study showed that LNG-IUS is very effective in providing symptomatic relief to adenomyotic patients, thereby improving the quality of life of the individuals. In patients who have increased UCL or high uterine volumes, there is a risk of displacement or expulsion of LNG-IUS, which can be overcome by hysteroscopic suture fixation inside the uterus.

## Figures and Tables

**Figure 2:**
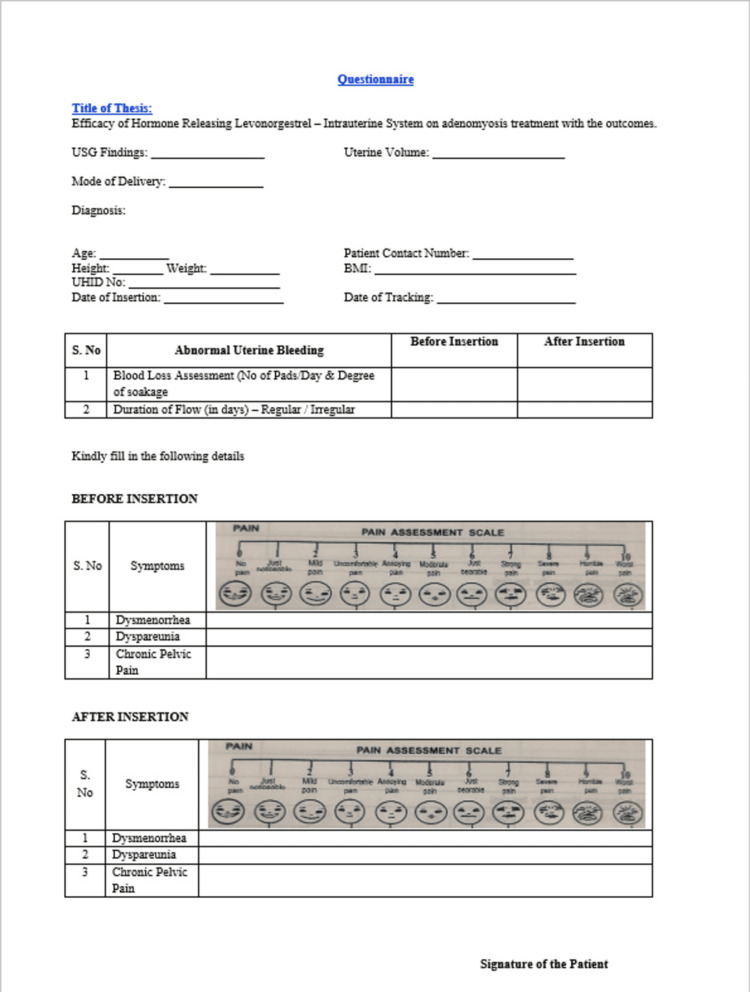
Study questionnaire.
